# Narrower QRS may be enough to respond to cardiac resynchronization therapy in lightweight patients

**DOI:** 10.1007/s00380-019-01541-8

**Published:** 2019-11-27

**Authors:** Toshiko Nakai, Hiroaki Mano, Yukitoshi Ikeya, Yoshihiro Aizawa, Sayaka Kurokawa, Kimie Ohkubo, Koichi Nagashima, Ichiro Watanabe, Yasuo Okumura

**Affiliations:** Department of Medicine, Division of Cardiology, Nihon University School of Medicine, 30-1 Oyaguchi Kamicho, Itabashi-ku, Tokyo 173-8610 Japan

**Keywords:** Body size, Left ventricular diameter, Conduction disturbance, CRT response

## Abstract

A prolonged QRS duration (QRSd) is promising for a response to cardiac resynchronization therapy (CRT). The variation in human body sizes may affect the QRSd. We hypothesized that conduction disturbances may exist in Japanese even with a narrow (< 130 ms)-QRS complex; such patients could be CRT candidates. We investigated the relationships between QRSd and sex and body size in Japanese. We retrospectively analyzed the values of 338 patients without heart failure (HF) (controls) and 199 CRT patients: 12-lead electrocardiographically determined QRSd, left ventricular diastolic and systolic diameters (LVDd and LVDs), body surface area (BSA), body mass index (BMI), and LVEF. We investigated the relationships between the QRSd and BSA, BMI, and LVD. The men’s and women’s BSA values were 1.74 m^2^ and 1.48 m^2^ in the controls (*p* < 0.0001), and 1.70 m^2^ and 1.41 m^2^ in the CRT patients (*p* < 0.0001). The men’s and women’s QRSd values were 96.1 ms and 87.4 ms in the controls (*p* < 0.0001), and 147.8 ms and 143.9 ms in the CRT group (*p* = 0.4633). In the controls, all body size and LVD variables were positively associated with QRSd. The CRT response rate did not differ significantly among narrow-, mid-, and wide-QRS groups (83.6%, 91.3%, 92.4%). An analysis of the ROC curve provided a QRS cutoff value of 114 ms for CRT responder. The QRSd appears to depend somewhat on body size in patients without HF. The CRT response rate was better than reported values even in patients with a narrow QRSd (< 130 ms). When patients are considered for CRT, a QRSd > 130 ms may not be necessary, and the current JCS guidelines appear to be appropriate.

## Introduction

Cardiac resynchronization therapy (CRT) is effective in some patients with heart failure (HF) [1, 2]. The use of CRT improves patients' functional capacity and quality of life, and it also decreases mortality among patients who have an intraventricular conduction disturbance in addition to their HF; patients with a QRS duration (QRSd) ≥ 150 ms in particular are promising candidates for CRT [3–5]. The European Society of Cardiology (ESC) guidelines [6] for CRT were revised in 2016 on the basis of fairly recent clinical trials [7, 8]. According to the new guidelines, a QRS complex ≥ 130 ms and sinus rhythm together comprise a Class I indication for CRT; a QRS complex < 130 ms is a Class III indication. These ESC guidelines seemed to have contributed to the spread of CRT in Japan. However, the Japanese Circulation Society (JCS) guidelines [9] consider a QRS ≥ 120 ms a Class I indication, and we have encountered many CRT responders not only among patients with a narrow QRS complex (i.e., a QRS < 130 ms) but even among patients with a very narrow QRS complex (< 120 ms) [10].

The sizes of human bodies vary considerably, and the QRS duration (QRSd) may depend to some degree on body size. Japanese are generally physically small, and based on our speculation that the QRSd depends in part on body size, we hypothesized that conduction disturbances can exist in Japanese even among those with a QRS complex < 120 ms. We conducted the present retrospective study to clarify the relationship between QRSd and body size in Japanese to evaluate the appropriateness of the JCS QRS-specific indications for CRT.

## Patients and methods

### Control group

The study population was comprised of patients at our hospital who had been referred to us for echocardiographic screening and for whom the echocardiographic variables were shown to be within the normal ranges. We reviewed the medical records of 569 patients (median age 66 years, range 27–98 years) without HF who underwent cardiac disease screening by means of both 12-lead electrocardiography (ECG) and echocardiography at Itabashi University Hospital in January 2017. Of the 569 patients, 231 were excluded from the study due to their complete right bundle branch block, organic heart disease, Wolff–Parkinson–White syndrome, or Brugada syndrome, and thus our final control group was 338 patients. From the medical records, we obtained the patients' ECG-determined QRSd value and height and body weight (BW), from which we calculated the body surface area (BSA) and body mass index (BMI) for each patient.

### CRT group

We examined the cases of the 241 consecutive patients (median age 67 years, range 14–88 years) who underwent CRT implantation at our institution during the period from October 2004 to January 2019. Patients who were upgraded to CRT from a pacemaker with right ventricular pacing (*n* = 42) were excluded, and thus the final study population of CRT patients was 199. The study protocol was reviewed and approved by the ethics committee of our institution.

Apical two- and four-chamber views were used to determine the left diastolic and systolic diameters (LVDd and LVDs), and the left ventricular ejection fraction (LVEF) was also obtained. We looked at differences between the males and females in these three clinical variables, and we investigated the relationships between the QRSd and these variables in both the control group and the group of CRT patients.

We divided the 199 CRT patients into three groups based on their QRSd values: the narrow group with a QRSd < 130 ms, the mid-group with a 130 ≤ QRSd < 150 ms, and the wide group with a QRS ≥ 150 ms. We ascertained the CRT response rate in each group. We defined ‘CRT response’ as a functional and echocardiographic response. We defined improvement in ‘functional status’ as a > 1 grade decrease in NYHA class at 6 months of follow-up. ‘Echocardiographic response’ was defined as a reduction in the left ventricular end-systolic volume (LVESV) of ≥ 15% or a reduction in the LVEF of ≥ 5% at 6 months after the CRT implantation.

Continuous variables are expressed as the mean ± SD. Differences between male and female patients were analyzed by *t* test or *χ*² test, as appropriate. The relationships between the QRSd and clinical variables, i.e., sex, BSA, BMI, LVDd, LVDs, and LVEF were assessed on the basis of Spearman's correlation coefficient. We performed a multiple regression analysis to determine which variables were related to QRSd. A receiver operating characteristic (ROC) curve was plotted to determine the cutoff value of QRSd for a response to CRT. All statistical analyses were performed with JMP 12.2.1 software (SAS Institute, Cary, NC, USA), and *p* values < 0.05 were considered significant.

## Results

Table 1 summarizes the clinical characteristics of the control group for the total series of 338 patients and for the two genders. The male/female ratio was 178/160. The patients’ mean age was 66.4 ± 14 years and did not differ significantly between the male and female patients. The BSA and BMI values differed significantly between the male and female patients, as did the other variables examined. The QRSd was significantly longer in the male patients than in the female patients at 96.1 ms versus 87.4 ms, respectively (*p* < 0.0001). The LVDd and LVDs values were significantly greater in the male patients than in the female patients at 48.8 mm and 31.1 mm versus 43.8 mm and 26.2 mm, respectively (*p* < 0.0001). The LVEF values were significantly greater in the females compared to the males at 70.9 ± 7.9% versus 65.2 ± 12%, respectively (*p* < 0.0001).

**Table 1:** Clinical characteristics of the patients and per sex in the control group

	Total (*n* = 338)	Male (*n* = 178)	Female (*n* = 160)	*p* value
Age, years	66.4 ± 14	65.3 ± 13	67.6 ± 14	0.1173
BSA, m^2^	1.61 ± 0.2	1.74 ± 0.16	1.48 ± 0.15	< 0.0001
BMI, kg/m^2^	23.2 ± 3.8	23.8 ± 3.6	22.6 ± 3.8	0.0023
QRS duration, ms	92 ± 11	96.1 ± 11	87.4 ± 9	< 0.0001
LVDd, mm	46.5 ± 6.5	48.8 ± 6.4	43.8 ± 5.6	< 0.0001
LVDs, mm	28.7 ± 6.9	31.1 ± 7.5	26.2 ± 5.2	< 0.0001
LVEF, %	67.9 ± 11	65.2 ± 12	70.9 ± 7.9	< 0.0001

Values are mean ± SD

*BSA* body surface area, *BMI* body mass index, *LVDd* left ventricular diastolic diameter, *LDVs* left ventricular systolic diameter, *LVEF* left ventricular ejection fraction

The correlations between the QRSd and each of the variables studied are shown in Table 2. Significant correlations were revealed between the QRSd and the patients' height, BW, BSA, LVDd, and LVDs values.

**Table 2:** Correlation between QRSd and study variables in the control group

Variable	Correlation coeff.^a^	*p* value
Age, years	0.0920	0.0913
BSA, m^2^	0.3338	< 0.0001
BMI, kg/m^2^	0.1895	0.0005
LVDd, mm	0.3582	< 0.0001
LVDs, mm	0.3711	< 0.0001
LVEF, %	0.2816	< 0.0001

Abbreviations are explained in Table 1 footnote

^a^Spearman’s r

The clinical characteristics of the 199 CRT patients and those for each gender are summarized in Table 3. The male/female ratio of the CRT group was 158/41; the mean age was 65.6 ± 13 years and did not differ significantly between the males and females. Variables pertaining to body size (i.e., BSA and BMI) differed significantly between the male and female patients, but no other variables showed significant differences between the males and females. The QRSd values were 147.8 ms and 143.9 ms for the males and females in the CRT group (*p* = 0.7683).

**Table 3:** Clinical characteristics of patients and per sex in the CRT group

	Total (*n* = 199)	Male (*n* = 158)	Female (*n* = 41)	*p* value
Age, years	65.6 ± 13	65.0 ± 12	67.6 ± 16	0.1713
BSA, m^2^	1.64 ± 0.2	1.70 ± 0.19	1.41 ± 0.15	< 0.0001
BMI, kg/m^2^	22.6 ± 4.4	22.9 ± 4.3	21.1 ± 4.6	0.0141
QRS duration, ms	147 ± 31	147.8 ± 31	143.9 ± 4.0	0.4633
LVDd, mm	65.9 ± 8.3	66.2 ± 8.2	64.1 ± 8.8	0.4430
LVDs, mm	56.2 ± 8.5	56.0 ± 8.5	52.5 ± 8.3	0.1934
LVEF, %	26.6 ± 89.1	26.3 ± 8.3	28.1 ± 13	0.4830
NYHA class	3.0 ± 0.53	3.02 ± 0.54	2.95 ± 0.50	0.4624

Values are mean ± SD

The LVDd and LVDs data in the CRT group were not significantly different between the males and the females at 66.6 mm and 58.0 mm versus 65.1 mm and 54.8 mm, respectively (*p* = 0.6724, *p* = 0.7561). The correlations between the QRSd and each of the variables studied are provided in Table 4. In the CRT group, there was no significant correlation between the QRSd and the BSA, MBI, LVDd, or LVDs values.

**Table 4:** Correlation between QRSd and study variables in the CRT group

Variable	Correlation coeff.^a^	*p* value
Age, years	0.09892	0.1645
BSA, m^2^	0.03631	0.6107
BMI, kg/m^2^	0.01162	0.8706
LVDd, mm	0.01656	0.8848
LVDs, mm	0.12563	0.3562
LVEF, %	0.15610	0.3574

^a^Spearman’s r

**Fig. 1:**
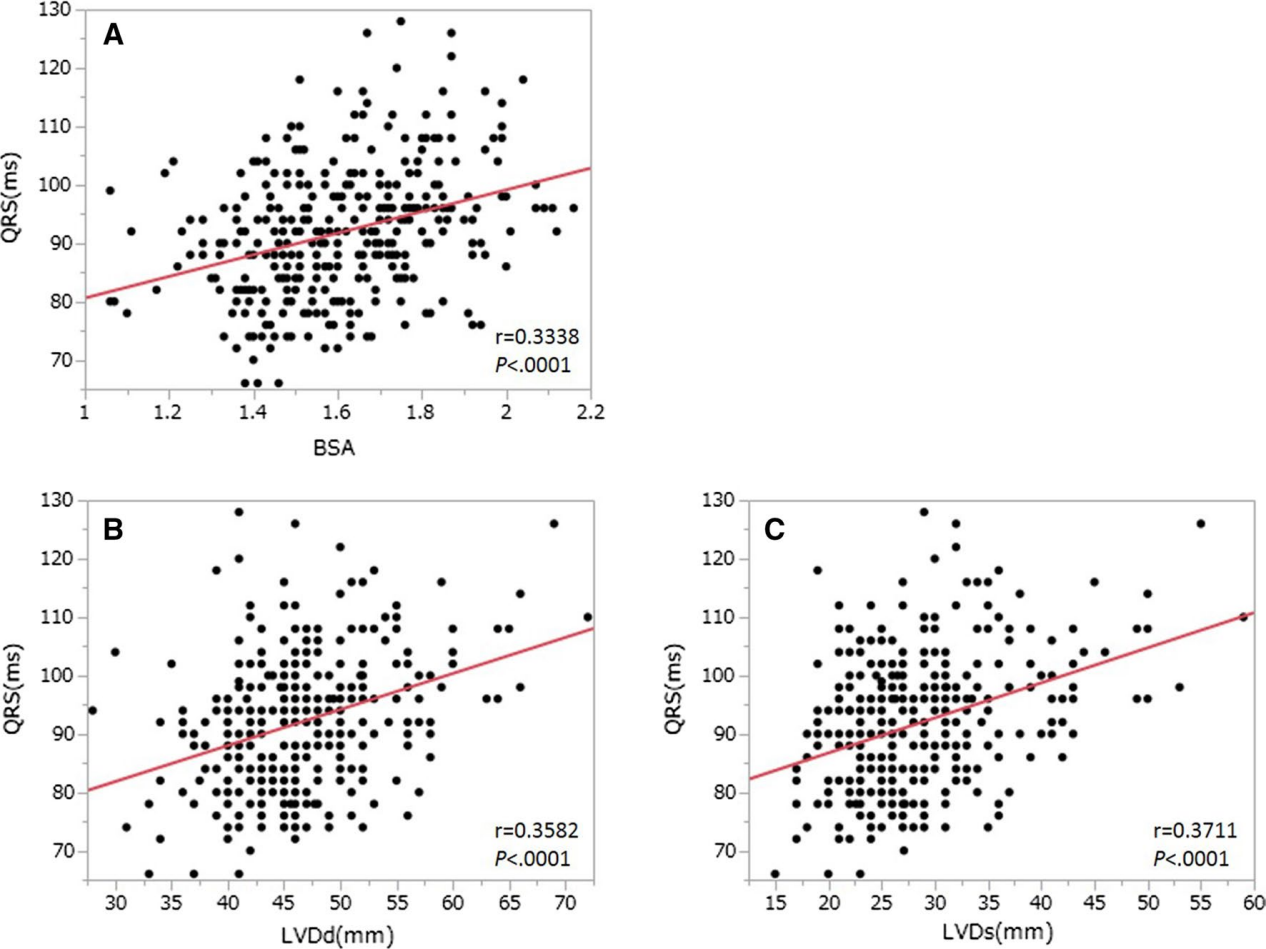
Scatterplots of the correlations between the QRSd and the BSA and the LVD variables. The correlations between the QRSd and **a** BSA, **b **LVDd, and **c **LVDs are positive

As noted above, the QRSd differed significantly between the male and female patients (Table 1). A significant correlation was observed between the QRSd and the variables examined with the exception of age; in addition, all variables that pertain to body size (i.e., height, BW, BSA, BMI, and LVDd and LVDs) correlated positively with the QRSd (Table 2). The results of the multiple regression analysis demonstrated that the CRT patients’ BSA was a strong determinant of the QRSd (*t* = 3.65, *β* = 0.27, *p* = 0.0003). The QRSd was shown to correlate positively with the BW, BSA, LVDd, and LVDs results (Table 2, Fig. 1a–c). However, there were no significant differences between the genders in these variables except for BSA (Table 3), and no correlation was identified between body size and the QRSd in the CRT patients (Table 4).

**Fig. 2:**
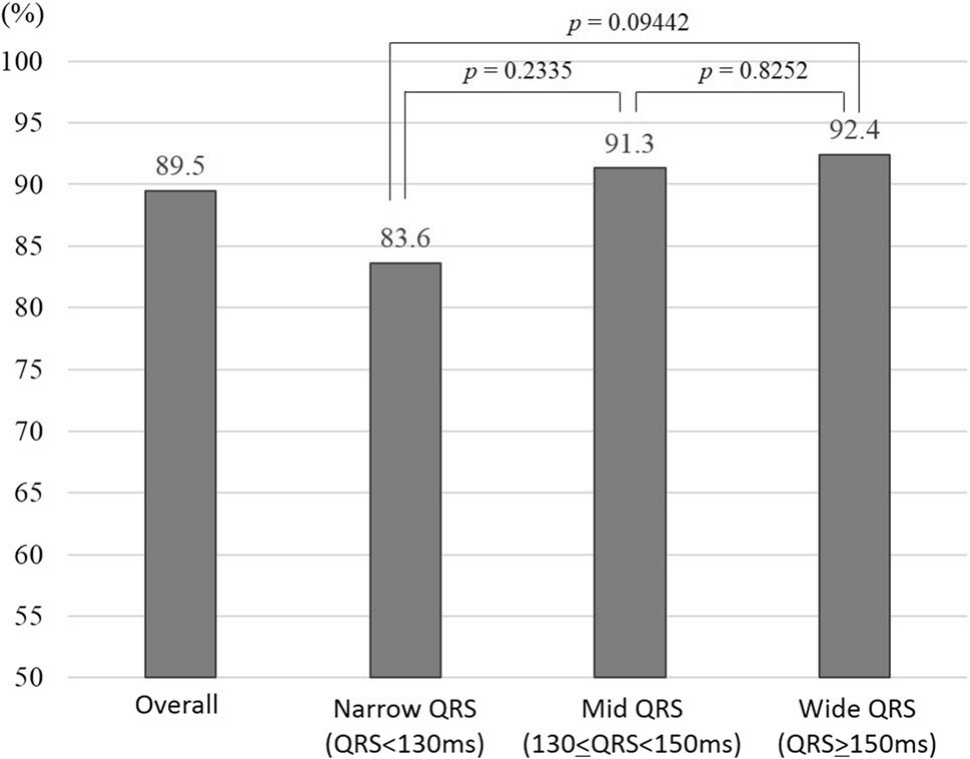
The CRT response rates of the overall patients and the narrow-, mid-, and wide-QRS groups. There were no significant differences in CRT response among the narrow, mid-, and wide groups. Narrow QRS: QRSd < 130 ms. Mid-QRS: 130 ≤ QRSd < 150 ms. Wide QRS: QRSd ≥ 150 ms

The overall CRT response rate of the CRT patients was 89.5%, which is better than the CRT response rates reported worldwide [1, 2, 4, 11], and there was no significant difference in the CRT response rate among the present narrow-, mid-, and wide-QRS groups (83.6%, 91.3%, and 92.4% respectively) (Fig. 2).

**Fig. 3:**
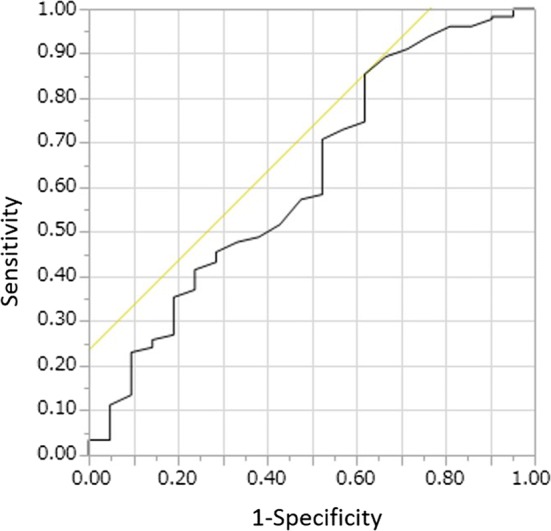
The ROC curve for predicting CRT responders. The QRSd of 114 ms was a cutoff for predicting CRT responders, with an area under the curve (AUC) of 0.62

An analysis of the ROC curve provided a QRSd cutoff value of 114 ms for responding to CRT, with an area under the curve (AUC) of 0.62, 85% sensitivity, and 38% specificity (Fig. 3).

## Discussion

The most important finding of this study was the significant difference in the QRSd between the men and women without HF. This difference reflects the difference in body size between men and women, including the difference in heart sizes. The correlation that we observed between the QRSd and the LVD measures could mean that the conduction time is associated with the LV size in patients without HF.

An association between the ventricular volume and the BSA has been reported, and a difference was observed in the ventricular volume between the sexes [12]. Body size is known to differ not only between males and females but also among the world's regions, with the BW of Japanese being less, on average, than that of Westerners [13, 14]. Accordingly, we expected the QRSd values of Japanese to be shorter than those of Westerners and, by extension, we suspected that the indication for CRT in Japanese patients would be a shorter QRSd. Interestingly, several studies in which responses to CRT have been assessed in relation to the patients' sex have shown better responses to CRT among women than among men [15–20]. Our present investigation did not reveal a significant difference in the CRT response between the men and women. This result might be due to the small number of women patients, or due to our inclusion of more advanced HF in the female patients. However, we speculate that our present findings can explain this sex-based difference in the response to CRT.

We suspect that it might be that the baseline QRSd in women who present with a wide QRS complex > 130 ms (making them candidates for CRT) is relatively short in comparison to the baseline QRSd in men who, as candidates for CRT, present with a similarly wide QRS complex. Karaca et al. showed that adjusting the QRS duration by the patient's BMI may contribute to the identification of appropriate candidates for CRT. They used a QRS index (QRSd/BMI) to evaluate their patients' CRT responses, and they reported that 5.5 ms/m^2^/kg as the cutoff value for the QRS index could be used to identify a CRT response [21]. In our present group of CRT patients, the QRS index was not correlated with the left ventricular end-diastolic volume (LVEDV)/LVESV (*r* = 0.0384, *p* = 0.7631/*r* = 0.02332, *p* = 0.8573, respectively), although the average LVEDV and LVESV values were 218.2 mL and 159.8 mL, which are as large as those of the CRT patients in the Karaca et al. study (211.8 mL and 156.7 mL). This suggests that the QRS and the QRS index do not always precisely reflect the enlargement of a patient's left ventricle, or they may underestimate the existence of conduction disturbances in Japanese patients with heart failure.

According to the ESC guidelines issued in 2016, a QRSd > 130 ms is ranked as a Class I indication for CRT, but a QRSd < 130 ms is ranked as a Class III indication. We Japanese physicians were surprised by these new guidelines because we have encountered many patients with a narrow QRS complex of 120–130 ms who have responded to CRT. We have also had patients with an even narrower QRS complex of < 120 ms whose conditions improved dramatically after CRT [9]. These patients were relatively thin, i.e., they had very little body fat, so given that their baseline QRSd was short, a ventricular disturbance could exist even with a QRSd of 110–120 ms. It might thus be too late if we wait until the QRSd becomes prolonged to > 130 ms in small patients; those with a narrower QRSd could be considered candidates for CRT, otherwise we may miss the optimal timing for CRT implantation. A too-severe HF status (NHYA class IV) is the most important predictor of mortality [22], and physicians must, therefore, be very careful to start therapy at an early stage and implant CRT at the appropriate time point, before patients progress to the advanced stage of HF.

However, special attention should be paid to the programming when we treat patients with a narrow QRS complex because the atrioventricular delay is very important and is critical for a response to CRT in these patients in particular. CRT has spread slowly in Japan because of the ESC guidelines naming a QRSd < 130 ms as a class III indication. We believe there is a real need to validate our JCS guidelines to save as many HF patients as possible; opportunities for heart transplantation are limited in Japan, especially in comparison to opportunities in many other countries. Since 1999, < 400 heart transplants have been performed in Japan, with 44 being the maximum number performed in a single year [23], and CRT is the most feasible means of saving the lives of patients with drug-resistant HF. The situation in Japan is very different from that in other countries.

Overall, our data indicate that Japanese patients with relatively small body sizes have a QRSd that is shorter than that of Westerners. A Japanese patient may have a severe conduction disturbance even if his or her QRSd is < 130 ms. Lightweight Japanese and other patients with a QRS complex < 130 ms may be good candidates for CRT. We believe that different guidelines should exist for Asians and Westerners and for men and women.

### Study limitations

Our study data should be interpreted in light of the following study limitations. First, although we excluded patients with complete right bundle branch block or another disorder resulting in a conduction delay from the study, it is possible that a disorder affecting ventricular conduction had gone undiagnosed in one or more of our study patients. Second, because there are currently limited published data concerning the normal average QRSd in Westerners, we were unable to make a direct comparison of the QRSd in Japanese and Westerners. What we did find was a clear difference in the QRSd between male and female Japanese patients without HF, and this difference was consistent with the differences in body size and heart size between men and women. Finally, women accounted for a small number of the CRT patients in this study, and the sex difference was not significant. However, the CRT response rate was higher than that reported worldwide even in patients with a narrow QRS complex, suggesting that a conduction disturbance can exist in Japanese patients even if their QRS complex is narrow at < 130 ms, and these individuals could be candidates for CRT.

## Conclusions

The QRSd is related to body size, and considering that the CRT response rate is relatively high in Japanese patients with a narrow QRS complex, the guidelines should be adjusted according to body size when considering the use of CRT for small patients such as the Japanese.
